# Pathological Findings in Autopsies Held on Five Cases during the Recent Yellow Fever Outbreak in San Antonio

**Published:** 1903-12

**Authors:** T. P. Lloyd


### Pathological Findings in Autopsies Held on Five Cases During the Recent Yellow Fever Outbreak in San Antonio.

BY T. P. LLOYD, M. D.

*Editor Texas Medical Journal:*

I have been asked to recite the finding at post mortem examinations in some of the cases of yellow fever who died in San Antonio. To rehearse them all would take up too much space, so I will only direct attention to four or five:

First. A Mexican, treated by Drs. Adolph Herff and John Herff, who made the diagnosis of yellow fever by the clinical course of the disease, and excluding malaria by careful and repeated blood examinations. The post mortem findings were as follows:

Man, age about 40, weight 160, well nourished, skin lemon yellow, sclera yellow, liver diminished in size, yellow, margins sharp; on section it showed decided fatty degeneration; gall bladder empty; spleen rather smaller than the average and dark in color. Stomach, mucous membrane decidedly ecchymotic at pylorus; duodenum also ecchymotic to the extent of about six inches below pylorus. Kidneys hyperemic. Stomach contained about six ounces of dark grumous fluid, containing disintegrated blood.

Next, Mrs. Oliver. Treated by Dr. A. S. McDaniel, who made a diagnosis of yellow fever, excluding malaria by blood examination. She was rather fleshy, age 68, weight about 180 pounds. Skin and sclerotics decidely icteric. Pupils dilated, gums bloody.

Liver about normal size, of yellowish color, friable and bloodless. Microscopic examination showed fatty degeneration to such a marked degree that the liver cells were hardly recognizable. Gall bladder empty. Spleen markedly smaller than normal, very dark in color. Stomach contained dark blackish fluid. Mucous membrane anæmic, showing decided ecchymosis and numerous eroded surfaces, duodenenal mucous membrane also ecchymotic. Kidneys enlarged and showed signs of recent inflammation. Heart muscles very friable and showed fatty degeneration.

Mr. J. P. Vance. Treated by Dr. M. J. Bleim, and diagnosed yellow fever, malaria being excluded by repeated and careful blood examinations. The cadaver presented the following picture a short time after death: Skin and sclerotics a very decided lemon yellow, pupils dilated, gums bloody, *rigor mortis* marked, ecchymotic spots on back of neck, sides of face, shoulders, lumbar regions, dependent portions of leg and thighs. Liver about normal size, retracted from ribs, very yellow color, friable and contained no blood, and showed extensive fatty degeneration. Gall bladder contained one-half ounce of dark, thick tarry fluid. Spleen was slightly enlarged and dark in color. Stomach contained several ounces of black fluid; mucous membrane pale, except for extensive ecchymosis; duodenal mucous membrane also decidedly ecchymotic. Right kidney had a large hemorrhagic area under capsule; it was decidedly hyperemic, and showed signs of recent inflammation.

Miss Groathans. Treated by Dr. C. M. Decker, who, from the clinical course of the disease, diagnosed the case one of yellow fever. Skin and sclerotics decidedly icteric, *rigor mortis* marked. Liver decided boxwood color, friable and bloodless; showed marked fatty degeneration. Gall bladder contained about three drachms of dark fluid. Spleen normal in size. Stomach contained several ounces of black, tarry fluid. Mucous membrane of stomach showed extensive ecchymotic areas, most marked near pylorus. Mucous membrane of duodenum was also very ecchymotic. Kidneys decidedly hyperemic, and showed signs of acute inflammation. Heart muscles friable.

The above mentioned cases were selected because they were well marked and presented all the pathological findings characteristic of yellow fever, and the diagnosis of which was unquestioned by all the doctors who witnessed them with the exception of two or three, who vainly tried to account for their death in some other way, arguing that pernicious malaria would cause the same pathological changes, or that acute yellow atrophy of the liver was the cause, complicated with nephritis, as in the case of Mrs. O., which necropsy was witnessed by more than forty of our best medical men, among whom was Geo. R. Tabor, Dr. Ferdinand Herff, dean of the medical profession of San Antonio, and a man of far wider experience than any other member of the profession here; Dr. Adolph Herff, Dr. F. Paschal, Dr. Lankford, Dr. Hicks, Dr. Watts, Dr. Shropshire, Dr. Jackson, Dr. Russ, Dr. Bliem, Dr. Clifford, Dr. Decker, Dr. Moss, Dr. Chapman, Dr. Dupuy and others whose names I can not at this moment recall.

Another diagnosis suggested was pernicious malaria "fulminated" by dengue, the combination of which, if that is what the gentleman means by "fulminated," is enough to cause most any kind of an "explosion," and we can readily see why his "fulminated" case burst so many blood vessels and bled so copiously from the nose.

Another "anti" doctor suggested that it was simply a fatal case of "janders."

